# Equity and Accessibility of Washington State’s COVID-19 Digital Exposure Notification Tool (WA Notify): Survey and Listening Sessions Among Community Leaders

**DOI:** 10.2196/38193

**Published:** 2022-08-03

**Authors:** Tyler Jarvis Bonnell, Debra Revere, Janet Baseman, Rebecca Hills, Bryant Thomas Karras

**Affiliations:** 1 Department of Epidemiology School of Public Health University of Washington Seattle, WA United States; 2 Department of Health Systems & Population Health School of Public Health University of Washington Seattle, WA United States; 3 Office of Innovation & Technology State of Washington Department of Health Tumwater, WA United States

**Keywords:** COVID-19 exposure notifications, digital public health, health equity, mHealth, mobile health, mHealth equity, digital health tool, public health, surveillance, COVID-19, smartphone, health inequity, sociodemographic factor, epidemiology

## Abstract

**Background:**

In November 2020, WA Notify, Washington State’s COVID-19 digital exposure notification tool, was launched statewide to mitigate ongoing COVID-19 transmission. WA Notify uses the Bluetooth proximity–triggered, Google/Apple Exposure Notification Express framework to distribute notifications to users who have added or activated this tool on their smartphones. This smartphone-based tool relies on sufficient population-level activation to be effective; however, little is known about its adoption among communities disproportionately impacted by the COVID-19 pandemic or what barriers might limit its adoption and use among diverse populations.

**Objective:**

We sought to (1) conduct a formative exploration of equity-related issues that may influence the access, adoption, and use of WA Notify, as perceived by community leaders of populations disproportionately impacted by the COVID-19 pandemic; and (2) generate recommendations for promoting the equitable access to and impact of this novel intervention for these communities.

**Methods:**

We used a 2-step data collection process to gather the perspectives of community leaders across Washington regarding the launch and implementation of WA Notify in their communities. A web-based, brief, and informational survey measured the perceptions of the community-level familiarity and effectiveness of WA Notify at slowing the spread of COVID-19 and identified potential barriers and concerns to accessing and adopting WA Notify (n=17). Semistructured listening sessions were conducted to expand upon survey findings and explore the community-level awareness, barriers, facilitators, and concerns related to activating WA Notify in greater depth (n=13).

**Results:**

Our findings overlap considerably with those from previous mobile health equity studies. Digital literacy, trust, information accessibility, and misinformation were highlighted as key determinants of the adoption and use of WA Notify. Although WA Notify does not track users or share data, community leaders expressed concerns about security, data sharing, and personal privacy, which were cited as outweighing the potential benefits to adoption. Both the survey and informational sessions indicated low community-level awareness of WA Notify. Community leaders recommended the following approaches to improve engagement: tailoring informational materials for low-literacy levels, providing technology navigation, describing more clearly that WA Notify can help the community, and using trusted messengers who are already engaged with the communities to communicate about WA Notify.

**Conclusions:**

As digital public health tools, such as WA Notify, emerge to address public health problems, understanding the key determinants of adoption and incorporating equity-focused recommendations into the development, implementation, and communication efforts around these tools will be instrumental to their adoption, use, and retention.

## Introduction

### WA Notify

In the spring of 2020, public health authorities partnered with Google and Apple in the development of smartphone-based COVID-19 exposure notification (EN) tools that could be activated on Android and iPhone devices. Public health officials hoped that these tools would supplement public health measures—such as manual case investigation and contact tracing, masking mandates, guidance regarding physical distancing and limited social gatherings, and COVID-19 testing—to mitigate the spread of COVID-19 [1]. WA Notify uses the privacy-preserving Google/Apple EN Express framework to distribute Bluetooth proximity–triggered notifications to users who have added or activated this tool on their smartphones [2]. Digital EN tools send alerts to smartphone users to let them know that they have potentially been exposed to someone who has tested positive for COVID-19. To do this, digital EN users who test positive for COVID-19 (ie, index cases) verify their test results through the tool which anonymously alerts other users of their potential exposure.

Recent evidence suggests that these tools can help mitigate the spread of COVID-19 [3-5]. However, their performance relies, in part, on sufficient adoption by smartphone owners [6]. Identifying the challenges to adoption could inform strategies to increase activations and communicate the value of digital EN tools to improve retention. In particular, understanding the barriers and facilitators to adoption and use by communities disproportionately impacted by the COVID-19 pandemic [7-11] could help tailor marketing campaigns to promote adoption and increase the likelihood that all populations benefit from this new public health technology.

On November 30, 2020, WA Notify, the Washington (WA) State digital EN tool, was launched statewide [2]. Early in the development and testing phases for WA Notify, the WA State Department of Health (DOH) and the University of Washington School of Public Health entered into an interagency agreement to conduct an evaluation of WA Notify as a public health strategy to mitigate COVID-19 transmission. One aim of this evaluation sought to identify the barriers and facilitators to the statewide adoption and use of WA Notify and ascertain potential unintended impacts of this novel technology. Between its launch and the time period in which this study was conducted (from November 30, 2020, to September 05, 2021), WA Notify had been activated on over 2.4 million smartphones across the state, representing an adoption rate of 54.8% of the state adult population. Privacy constraints do not allow for users, user characteristics or demographics, or their location to be identified, so it is not possible to know whether the residents of communities most disproportionately impacted by COVID-19 were adopting or benefiting from WA Notify.

### Equity and Mobile Health Interventions

Health equity research focused on mobile health (mHealth) tools investigates how to address, simplify, and remove structural barriers to act upon and benefit from health information presented in public health interventions [12,13] and ensure that the technology does not create additional barriers for different subpopulations [14,15]. The wide adoption and penetration of smartphone ownership across diverse subpopulations [16,17] has supported a proliferation of smartphone-based tools, programs, and interventions. Along with this growth, there have been concerns that interventions delivered solely through these devices may result in inequalities that exacerbate, rather than mitigate, existing health inequities [13,15,18-21]. Approaches suggested to address these issues and encourage a wider adoption of mHealth tools include providing training and technical assistance, leveraging familial and community connections, partnering with trusted institutions, and offering users control over the levels of privacy and anonymity [18-24], as well as developing these tools using user-centered and participatory design processes that engage community members and other potential end users [21,25].

However, identifying the optimal design elements and implementation strategies that enable a novel mHealth tool to promote health equity requires time. The COVID-19 pandemic was unprecedented in scope and urgency. There was little time to engage in formal research, iterative design cycles, or systematic evaluations before the launch of WA Notify. Additionally, public health’s ability to conduct equity, accessibility, and usability studies were constrained by a rapidly shifting landscape and competing priorities such as COVID-19 vaccine distribution efforts [26]. Our work aimed to address this gap in understanding by speaking directly with community leaders about their experiences, concerns, and opinions about WA Notify.

## Methods

### Ethical Considerations

The project plan was reviewed by the University of Washington Institutional Review Board and determined to be a public health surveillance quality improvement activity that did not require human subjects research review.

### Sample

We sought to enroll community leaders that were engaged with community-based COVID-19 mitigation efforts and representative of the diversity of populations and regions across WA state. Information and outreach about the project were circulated to key stakeholders, organizations, and equity advocates with the assistance of the WA State DOH COVID-19 Vaccine Implementation Collaborative (VIC), a group working to ensure equitable COVID-19 vaccine access across the state [27]. VIC membership includes community-based organization (CBO) leaders; community and social service group advocacy representatives; tribal health, public health, and health care organization representatives,;and interested community members. These individuals were recruited because of their deep knowledge of their communities and their experience directly engaging community members around COVID-19 interventions including masking, vaccination, and WA Notify.

### Framework

**Figure 1:**
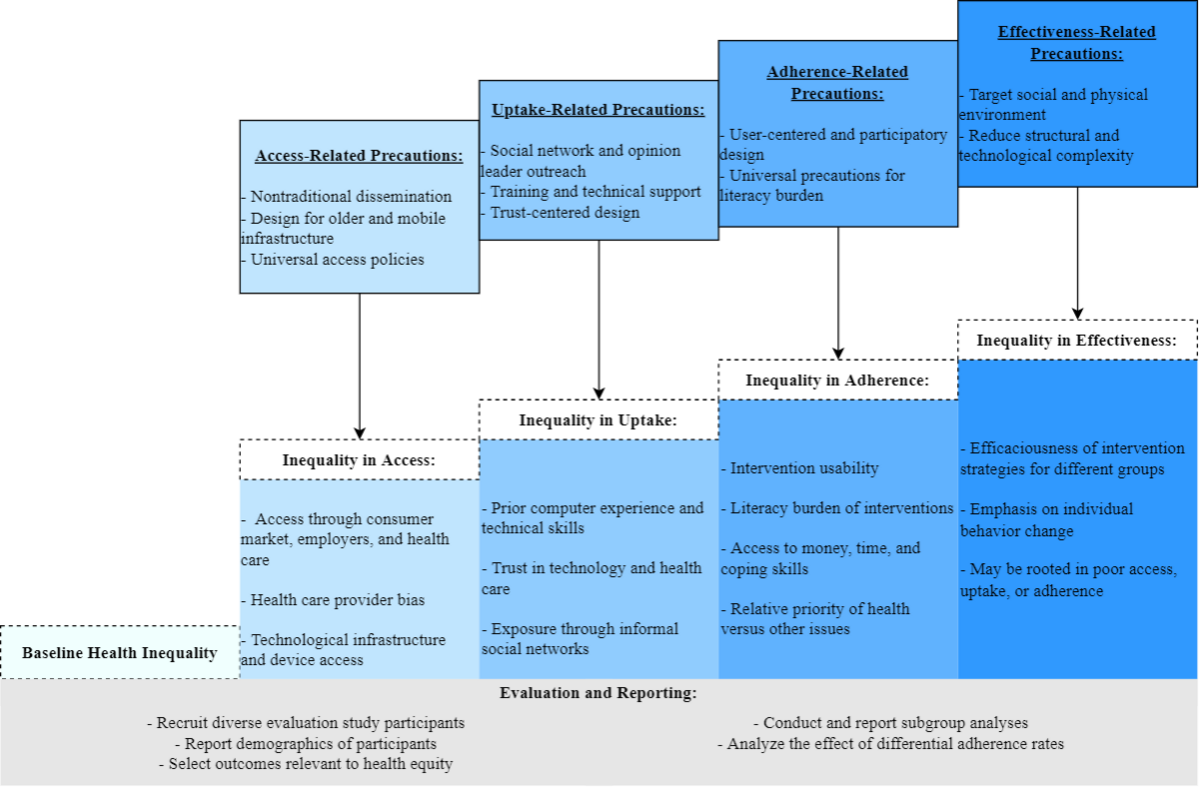
Intervention-Generated Inequality (IGI) Prevention Model adapted for clarity of presentation, used with permission of Oxford University Press (Veinot, et al) [18].

We used Veinot et al [18]’s model for intervention-generated inequality as a framework to inform our data collection efforts and contextualize our findings (Figure 1). This model proposes that inequalities in mHealth access, uptake, adherence, and effectiveness determine how these interventions influence pre-existing inequities [18]. Our data collection efforts primarily focused on investigating the potential access and uptake inequalities of WA Notify as the community-level adoption of this tool will influence its downstream effectiveness for diverse subpopulations. Additionally, we used this model to conceptualize how the issues and recommendations reported by participants influence the ability of this novel intervention to promote equitable health outcomes.

### Procedures

#### Exploratory Sequential Approach

For this evaluation, we used 2 data collection instruments: an informational survey followed by semistructured listening sessions. This exploratory sequential approach was chosen to quantify the levels of awareness, facilitators, barriers, and concerns with activating WA Notify across diverse settings and subsequently investigate the underlying community contexts that may influence the adoption of WA Notify. The 2 instruments were designed and used as follows.

#### Informational Survey

The survey was designed to take no more than 15 minutes to complete. In addition to gathering information regarding the respondent’s role or position in their community or organization, survey items focused on respondents’ description of conditions or contributors to the disproportionate impacts of COVID-19 on their communities (such as access barriers, underlying health conditions, and systemic inequities); the level of familiarity with WA Notify; perceptions regarding the effectiveness of WA Notify in limiting the spread of COVID-19; and the barriers and facilitators to the adoption and use of WA Notify by their community members.

The survey was programmed and distributed through the REDCap web-based platform (REDCap Consortium) [28] and available in hard-copy format upon request. The recruitment of CBO leaders and community advocates serving on the VIC was conducted by email in April 2021. Surveys closed in August 2021.

#### Semistructured Listening Sessions

The listening sessions were designed to capture details regarding the impact of COVID-19 in the participants’ community; awareness of WA Notify, how it works, and what communications have been received around its adoption and use; community concerns about using a digital EN tool; challenges and opportunities that might be leveraged to improve communications about WA Notify; and feedback regarding the best avenues for informing their communities about the value of using digital EN tools to protect oneself and loved ones.

**Figure 2:**
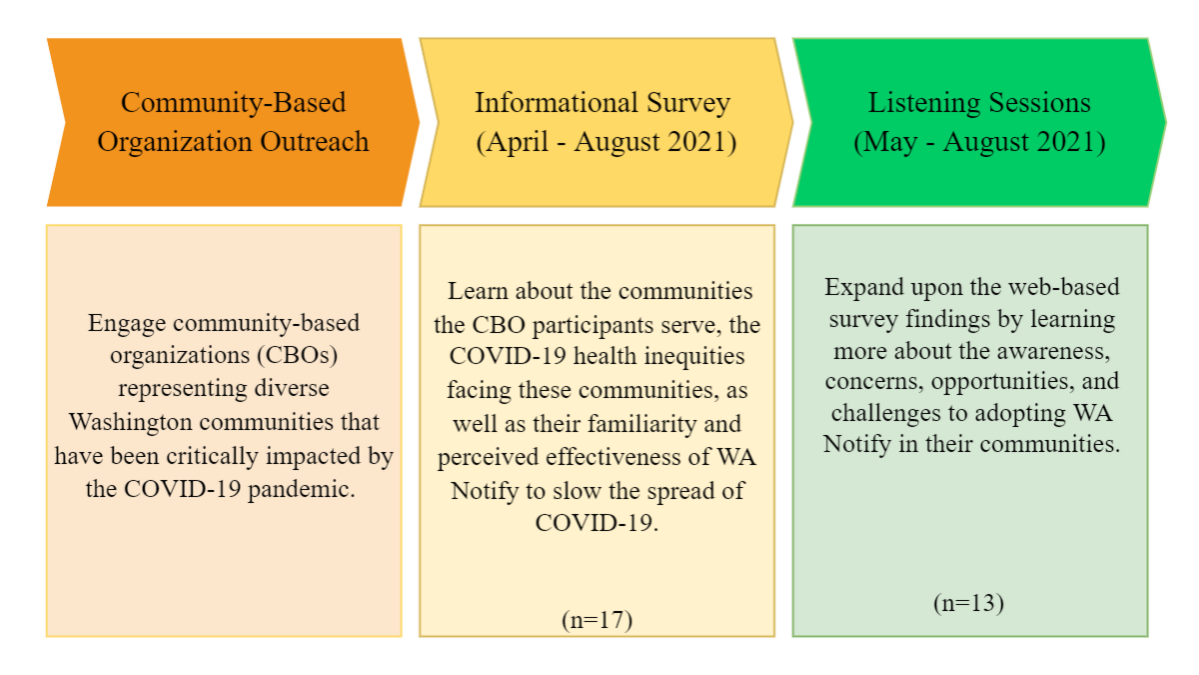
Equity and accessibility evaluation procedures.

Listening sessions were designed to last approximately 1 hour and were conducted remotely over Zoom software (Zoom Video Communications) at the participant’s convenience. Sessions were recorded to aid in notetaking with the participant’s permission. CBO leaders and community advocates who completed the informational survey were invited to participate in the listening sessions by email. The listening sessions were conducted between May and August 2021 (Figure 2).

### Analysis

Descriptive statistics were used to summarize the survey data. Listening session notes were qualitatively analyzed by 2 team members to identify emergent themes related to the intervention-generated inequality concepts of mHealth tool access, uptake, adherence, and effectiveness. Survey and listening session findings were synthesized to generate the results and recommendations for further outreach and communications that may improve the adoption and use of WA Notify in communities disproportionately impacted by COVID-19.

### Stakeholder Reflection

The synthesis of results and themes were circulated to DOH stakeholders and all participants in November 2021 to gather feedback on the results as well as capture any changes in perceptions on the adoption and use of WA Notify in participants’ communities. This input was incorporated into the thematic synthesis of results described in the next section.

## Results

### Participants

In our participant sample, 17 individuals completed the informational survey and 13 subsequently participated in listening sessions (Table 1). Community and CBO representation was broad, with participants from across the state and including CBO leaders and those advocating for or representing the following groups: older adults; low-income families; people with disabilities; essential and frontline workers; justice-impacted communities; individuals whose health care access is limited or impacted by stigma and bias such as individuals who identify as Lesbian, Gay, Bisexual, Transgender, Queer/Questioning, Asexual, and other identities (LGBTQIA+); American Indian or Alaska Native, Asian, Black or African American, Latinx, Muslim, and multiracial communities; immigrant, refugee, and undocumented communities; and rural and frontier communities (Table 1).

**Table 1:** Informational survey and listening session participants.

Represented communities^a^	Informational survey (n=17)	Listening session (n=13)
Essential workers and seniors with disabilities	✓	✓
Black, Indigenous, and People of Color; disabled; and justice-impacted communities	✓	✓
African American communities	✓	
Latinx communities, immigrants and refugees, and rural and frontier communities	✓	✓
Youth and seniors that cannot gain access to COVID-19 vaccines	✓	
Low-income families with young children, immigrants, and refugees	✓	✓
Amharic-speaking communities	✓	✓
Undocumented individuals and Latinx communities	✓	✓
Arabic-speaking immigrants and refugee	✓	✓
Muslims communities, individuals whose health care is impacted by stigma and bias, and LGBTQIA+^b^ communities	✓	✓
Latinx communities	✓	✓
Asian American Pacific Islander, Black, Indigenous, Latinx, and multiracial communities	✓	✓
People with disabilities	✓	
Immigrants and refugees from Arabic countries (Afghanistan, Iran, Iraq, Somalia, India, and Pakistan)	✓	
Essential and frontline workers	✓	✓
Blind, low-vision, and deafblind communities with various intersectional identities	✓	✓
Low-income older adults and people with disabilities	✓	✓

^a^Overlap and intersectionality among the communities represented listed above reflects the descriptions provided by participants.

^b^LGBTQIA+: Lesbian, Gay, Bisexual, Transgender, Queer/Questioning, Intersex, Asexual, and other identities.

### WA Notify Awareness

Participants perceived that the awareness and adoption of WA Notify was low in their communities. In the listening sessions, 1 participant who advocates for justice-impacted communities pointed out how they had not heard about WA Notify outside of their engagements with the DOH’s VIC, whereas most other participants mentioned only seeing communications during WA Notify’s initial launch in the fall of 2020. Participants also reported that without a basic understanding of what WA Notify is and how it works, adoption and use would be unlikely. For community members who wish to learn more about WA Notify, they currently need to actively seek information about the tool (via the DOH website, fact sheets, or videos), which was perceived as a barrier. Promotion by community leaders was identified as a key strategy to elevate community awareness; however, participants stated that unless community leaders themselves understand the “ins and outs” of WA Notify, they may be hesitant or ill-equipped to advocate in the community on its behalf.

**Figure 3:**
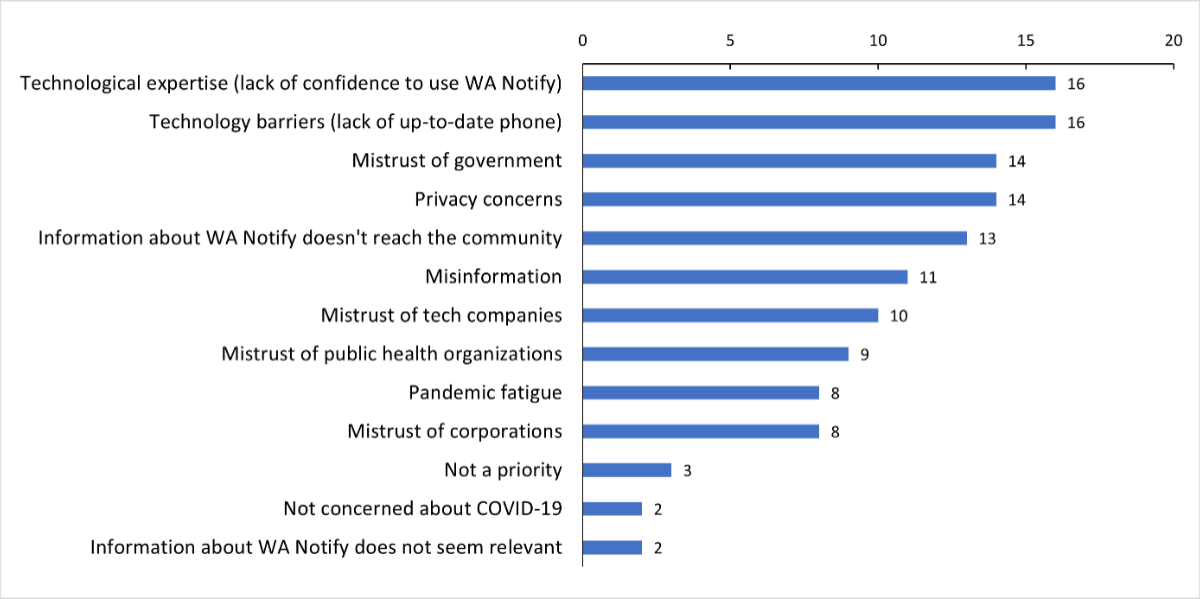
Perceived barriers to adding WA Notify to a smartphone by the communities represented by the survey respondents (N=17).

Possible contributors to the limited awareness and penetration of the tool were identified in the survey question, “Of these known barriers to adding WA Notify to a smartphone, which are relevant for your community?” (Figure 3) and further investigated during the listening sessions. The latter activity additionally captured suggested mechanisms or approaches for improving awareness, acceptance, and adoption.

### Thematic Synthesis

#### Themes

Digital expertise and confidence, privacy and security concerns, trust, and health and digital literacy levels emerged as key determinants of WA Notify awareness, acceptance, and adoption among vulnerable communities that might benefit from using WA Notify to help limit the spread of COVID-19 (Textbox 1).

**The 5 emergent themes after the synthesis of survey and listening session results.**

**Themes**

- Limited experience, expertise, and comfort with technologies, as well as access to smartphones, are substantial barriers to the adoption and use of WA Notify.
- Concerns about data security, tracking, and privacy outweigh or diminish the perceived value, benefits, and performance expectations of WA Notify for limiting the spread of COVID-19.
- The mistrust of government, law enforcement, and technology companies and the fear of negative consequences from engaging with exposure notification tools are substantial barriers to adoption.
- Language translations alone are not sufficient to ensure that WA Notify is accessible to communities with diverse health literacy, digital literacy, numeracy, and preferences for accessing information.
- Uptake may be improved by lowering literacy burdens, providing technology navigators, and disseminating tailored outreach and informational materials through trusted community social networks and opinion leaders.

#### Theme 1: Limited Experience, Expertise, and Comfort With Technologies, as Well as Access to Smartphones, Are Substantial Barriers to the Adoption and Use of WA Notify

The top 2 barriers to WA Notify adoption identified by survey respondents (N=17) concerned technology—the lack of an up-to-date phone (n=16, 94%) and lack of confidence to use a tool such as WA Notify (n=16, 94%; Figure 3). After exploring these issues further during our listening sessions, participants reported that a portion of their community members do not own or have access to a smartphone. In addition, CBO leaders working with immigrant and refugee communities noted a technology access divide based on gender and age, reporting that mobile phones are typically owned by men or household “breadwinners.”

Regarding community members who own or have access to a smartphone, there were concerns about the digital knowledge and skills required to use WA Notify. The process of adding or activating WA Notify requires several steps. Navigating through menus and downloading apps, even if the information is provided in the smartphone user’s native language, may be unfamiliar for those with limited digital skills.

#### Theme 2: Concerns About Data Security, Tracking, and Privacy Outweigh or Diminish the Perceived Value, Benefits, and Performance Expectations of WA Notify for Limiting the Spread of COVID-19

Survey respondents perceived privacy concerns (14/17, 82%) and misinformation (11/17, 65%) as barriers to adoption (Figure 3). Although WA Notify does not track its users or their locations, security and privacy were universal concerns reported during the listening sessions. In some communities, CBO leaders reported that misinformation about data privacy persists, perhaps due to a lack of information readily available to rectify this misperception or few opportunities for the community to present their questions or concerns. Additionally, an LGBTQIA+ community advocate highlighted that without a proper understanding of how WA Notify works, community members cannot fully discern the benefits it may provide them.

Related to Theme 1, to understand WA Notify’s benefit, some level of digital fluency is required to accept the premise that contact between 2 users is determined by proximity (via the exchange of randomized codes over Bluetooth), not location. Furthermore, unless communities understand what WA Notify is and how it works, its value in helping to protect community members and limit the spread of COVID-19 is not apparent.

#### Theme 3: The Mistrust of Government, Law Enforcement, and Technology Companies and the Fear of Negative Consequences Are Substantial Barriers to Adoption

Survey respondents identified the mistrust of government (14/17, 82%), tech companies (10/17, 59%), public health organizations (9/17, 53%), and corporations (8/17, 47%) as barriers to adoption within their communities (Figure 3). During the listening sessions, participants pointed out that the historical context between the government and their communities and the fear of repercussions influence the willingness to adopt an intervention such as WA Notify. Gaining trust in these communities from the “outside” is challenging. Related to Themes 1 and 2, in the absence of a clear understanding of how WA Notify works and how it can help both the individual user and their community without jeopardizing privacy and security, the risks of adoption outweigh the potential benefits.

**Figure 4:**
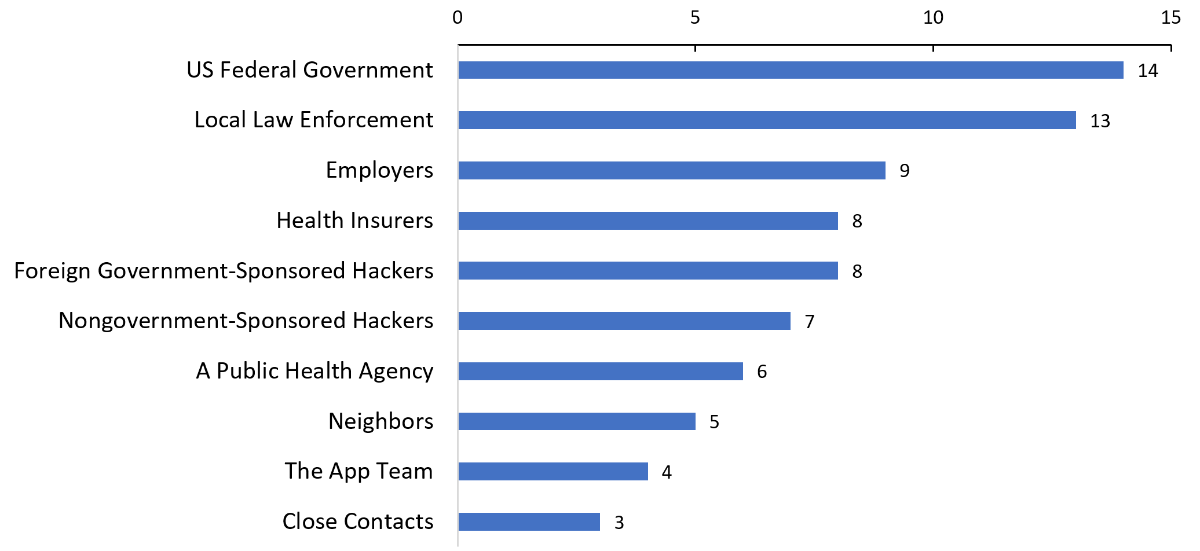
Entities of greatest concern to community members for accessing information through WA Notify (N=15).

When asked, “Who do you think members of your communities would be worried about having access to the information from the WA Notify tool?” respondents (N=15) identified the US federal government (n=14, 93%) and local law enforcement (n=13, 87%) as the entities of greatest concern (Figure 4).

Although WA Notify’s anonymous design appears to cater to the preferences of some communities to remain anonymous from the government, CBO leaders working with undocumented communities noted concerns that information could be collected by WA Notify and put individuals at risk of deportation. In communities that have seen cooperation between local government and the US Immigration and Customs Enforcement leading to deportation, this fear is especially prevalent. As noted by 1 participant, community members are always asking themselves, “Am I opening myself up to deportation by doing this?” In Afghani immigrant communities, CBO leaders reported that those with Special Immigrant Visas will avoid any activity, such as activating WA Notify, that could potentially jeopardize their residency status.

The negative consequences associated with employers (9/15, 60%) accessing information through WA Notify were also brought up in the listening sessions. Included in a WA Notify alert of possible exposure is public health guidance to engage in protective behaviors such as getting tested and quarantining (quarantine guidance during the time of this evaluation was 10 days). Participants noted that low-income community members may be particularly vulnerable to the consequences of receiving an EN and cannot risk losing multiple days of income. To activate WA Notify, these community members need a compelling answer to the question, “What am I getting out of this?” as well as assurance that comprehensive resources and support will be available to them upon receiving an EN.

#### Theme 4: Language Translations Alone Are Not Sufficient to Ensure That WA Notify Is Accessible to Communities With Diverse Health Literacy, Digital Literacy, Numeracy, and Preferences for Accessing Information

Listening session participants representing non–English-speaking, immigrant, and refugee communities repeatedly stated that the translation of materials into languages other than English is not an end point for ensuring equitable access to WA Notify. First, community members are often unaware that translated resources are available. In addition, even when translated, existing public health outreach materials and graphics may be difficult to understand for community members with lower levels of digital and health literacy. Multiple participants pointed out that current informational materials, while appropriate for individuals who are college educated, would need to be simplified to be appropriate for all members of their communities.

Listening session participants that advocate for diverse disability communities also identified the need to evaluate to what extent WA Notify is interoperable with critical assistive technologies (such as text-to-speech systems or screen readers) and identify strategies to ensure that the information and resources provided by this tool are accessible in a variety of formats.

#### Theme 5: Uptake May Be Improved by Lowering Literacy Burdens, Providing Technology Navigators, and Disseminating Tailored Outreach and Informational Materials Through Trusted Community Social Networks and Opinion Leaders

During the listening sessions, CBO leaders shared suggestions for improving the acceptance and adoption of WA Notify in their communities (Textbox 2).

**Participant recommendations for improving the acceptance and adoption of WA Notify.**

**Recommendations**

- Ensure that communication and informational materials are accessible at all literacy levels and work with assistive technologies
- Provide technology navigators
- Use trusted messengers who are known, credible, and already engaged in the community

##### Recommendation 1: Ensure That Communication and Informational Materials Are Accessible at All Literacy Levels and Work With Assistive Technologies

CBO leaders advocated for WA Notify and its promotional materials to use simple, easy-to-understand design elements. One CBO leader who had been providing Wi-Fi hot spots to immigrant and refugee families during the initial phase of the COVID-19 pandemic recalled needing to configure the devices to (1) limit the number of taps or clicks needed to access information; (2) use symbols to simplify navigation; and (3) provide a readily available technical support telephone line for troubleshooting. This participant emphasized that it is the responsibility of implementers, not the community members, to ensure that WA Notify is easy to understand and use.

##### Recommendation 2: Provide Technology Navigators

Activating WA Notify on a smartphone is difficult without technical or hands-on support. CBO leaders suggested that technical assistance be paired with existing community outreach activities, such as tabling sessions, workplace initiatives, and community health worker programs. CBO leaders also noted that for some families in their communities, the adult parent or guardian’s initial exposure to technology occurs through their children. For these communities, youths, particularly high school– and college-aged students, might be engaged as technology navigators due to their high digital literacy and familiarity with community networks. In addition, leveraging schools and libraries to provide technology assistance may help resolve some technology barriers. In summary, there is a need for interactive, low-barrier assistance to ensure that community members with limited digital skills are supported in activating and using WA Notify.

##### Recommendation 3: Use Trusted Messengers Who Are Known, Credible, and Already Engaged in the Community

Engaging trusted messengers was viewed as an effective means of circulating accurate information about WA Notify, especially for individuals not reached through previous communication efforts. Trusted messengers referenced during listening sessions include faith-based organizations, community health workers, health care providers, and CBO leaders. Providing information through these messengers could not only help elevate the awareness of WA Notify but may also address the dual challenges of mistrust and misinformation that limit adoption. One participant who advocates for immigrant and refugee communities articulated that their community members would likely ignore or scroll by WA Notify advertisements unless they were shared by a familiar organization. Engaging trusted messengers could help facilitate greater bidirectional communication between the DOH and communities regarding WA Notify, where trusted messengers could serve as nonjudgmental resources that directly engage with their community about their concerns and advocate for the requested changes to WA Notify.

## Discussion

### Principal Findings

This evaluation sought to uncover the equity and accessibility issues that may influence the access, adoption, and use of digital EN tools among diverse populations disproportionately impacted by COVID-19. Our findings suggest that digital literacy, trust, and information accessibility are key determinants of the adoption and use of WA Notify. Consistent with other mHealth equity evaluations of tools and interventions delivered over smartphones to disadvantaged and diverse populations, our results predominantly concerned limited health literacy, the ease of adoption and use, and issues related to the accessibility, relevance, and clarity of delivered content. However, misinformation and the fear of negative consequences related to using a digital EN tool emerged as unique determinants. These concerns may be barriers unique to a digital EN intervention or may be part of broader concerns among disenfranchised groups related to COVID-19 mitigation measures such as masking, contact tracing, and vaccination [29].

This evaluation also sought to generate recommendations from populations experiencing COVID-19–related health inequities to prevent WA Notify from contributing to the adverse effects of public health interventions and ensure that it is accessible, relevant, useful for diverse communities. To address inequalities in technology access and digital skills, we recommend providing technology navigation through social networks and as extensions of existing community-based programs. To prevent inequalities in adoption, we recommend expanding partnerships with trusted messengers to elevate awareness, address misinformation, and promote trust in this novel technology, especially among community members who may not have been engaged through previous communication efforts. To address inequalities in using or adhering to WA Notify, information should be simple, easy to navigate, and accessible in a variety of formats. Lastly, to prevent inequalities in effectiveness and ensure that digital EN tools actively narrow COVID-19–related health inequities, implementers need to identify critical structural barriers faced by vulnerable populations and develop strategies to help users address, navigate, or simplify these barriers. In essence, WA Notify should serve a dual purpose: (1) to notify individuals of a COVID-19 exposure and (2) provide comprehensive resource navigation to ensure that all users are able to engage in protective behaviors. By developing effective connections with CBOs, local health jurisdictions, and other social support or medical services, WA Notify could ensure greater benefits for disadvantaged individuals and maximize its potential impact against COVID-19–related morbidity and mortality.

### Limitations

This evaluation is subject to several limitations. First, although the project focused on identifying factors that could help WA Notify promote health equity, the direct measurement of the impact of WA Notify on COVID-19–related outcomes or health equity was not possible. Estimating the impact of digital EN systems is an ongoing area of evaluation, but the privacy-preserving design of this intervention does not allow for the identification of specific users, let alone their health status. Second, recruitment efforts were concentrated on a small, purposive sample of community leaders. Although these leaders provided invaluable insight into their communities, these individual perspectives are unlikely to capture the heterogeneity of lived experiences, insights, and recommendations of their communities or state. Additionally, leaders participating in both the survey and informational sessions identified themselves as representing more than one community, so it is not possible to know whether the perceptions they shared were those of one specific community. Subsequent phases of this evaluation will seek to engage community members directly to supplement and expand upon the insights shared by the community leaders. Lastly, our findings reflect the conditions experienced by diverse communities during the first year of WA Notify’s implementation. As the pandemic landscape changes, some of our results may no longer be generalizable to this new context. In the months following the completion of this evaluation, WA Notify has undergone iterative design changes, new variants (eg, Omicron) have emerged, and COVID-19 guidance has been modified. Future evaluations will need to take this altered landscape into account.

### Conclusion

WA Notify is more than just a digital EN tool. It is an intervention on an individual’s phone that represents public health and serves as a novel opportunity for public health agencies to communicate, alert, and engage with the communities they serve. WA Notify is part of a larger shift toward digital public health, which encompasses emerging tools such as vaccine verification initiatives and symptom-monitoring platforms, and seeks to improve the efficiency, scalability, and acceptability of traditional public health workflows. As an innovation that is changing how public health and outbreak response are conducted, WA Notify can empower its users to engage in the protective measures needed to keep themselves, their families, and their communities safe. Incorporating our equity-related findings and recommendations into the development, implementation, and communications efforts around digital pandemic and emergency preparation and response tools such as WA Notify will ensure that all communities can benefit.

## Abbreviations

CBO: community-based organization
DOH: Department of Health
EN: exposure notification
LGBTQIA+: Lesbian, Gay, Bisexual, Transgender, Queer/Questioning, Intersex, Asexual, and other identities
mHealth: mobile health
VIC: Vaccine Implementation Collaborative
WA: Washington
